# Stress-Inducible SCAND Factors Suppress the Stress Response and Are Biomarkers for Enhanced Prognosis in Cancers

**DOI:** 10.3390/ijms24065168

**Published:** 2023-03-08

**Authors:** Mona Sheta, Kunihiro Yoshida, Hideka Kanemoto, Stuart K. Calderwood, Takanori Eguchi

**Affiliations:** 1 Department of Dental Pharmacology, Faculty of Medicine, Dentistry and Pharmaceutical Sciences, Okayama University, Okayama 700-8525, Japan; 2 Department of Cancer Biology, National Cancer Institute, Cairo University, Cairo 11796, Egypt; 3 Department of Oral and Maxillofacial Surgery, Graduate School of Medicine, Dentistry and Pharmaceutical Sciences, Okayama University, Okayama 700-8525, Japan; 4 Department of Radiation Oncology, Beth Israel Deaconess Medical Center, Harvard Medical School, Boston, MA 02115, USA

**Keywords:** cell stress response, heat shock protein 90 (HSP90), SCAN domain (SCAND)-containing proteins, MZF1/ZSCAN6, heat shock factor (HSF), long noncoding RNA (lncRNA), co-expression correlation, Kaplan–Meier plot, cancer patient prognosis

## Abstract

The cell stress response is an essential system present in every cell for responding and adapting to environmental stimulations. A major program for stress response is the heat shock factor (HSF)–heat shock protein (HSP) system that maintains proteostasis in cells and promotes cancer progression. However, less is known about how the cell stress response is regulated by alternative transcription factors. Here, we show that the SCAN domain (SCAND)-containing transcription factors (SCAN-TFs) are involved in repressing the stress response in cancer. SCAND1 and SCAND2 are SCAND-only proteins that can hetero-oligomerize with SCAN-zinc finger transcription factors, such as MZF1(ZSCAN6), for accessing DNA and transcriptionally co-repressing target genes. We found that heat stress induced the expression of SCAND1, SCAND2, and MZF1 bound to *HSP90* gene promoter regions in prostate cancer cells. Moreover, heat stress switched the transcript variants’ expression from long noncoding RNA (lncRNA-SCAND2P) to protein-coding mRNA of SCAND2, potentially by regulating alternative splicing. High expression of *HSP90AA1* correlated with poorer prognoses in several cancer types, although SCAND1 and MZF1 blocked the heat shock responsiveness of *HSP90AA1* in prostate cancer cells. Consistent with this, gene expression of *SCAND2*, *SCAND1*, and *MZF1* was negatively correlated with *HSP90* gene expression in prostate adenocarcinoma. By searching databases of patient-derived tumor samples, we found that MZF1 and SCAND2 RNA were more highly expressed in normal tissues than in tumor tissues in several cancer types. Of note, high RNA expression of SCAND2, SCAND1, and MZF1 correlated with enhanced prognoses of pancreatic cancer and head and neck cancers. Additionally, high expression of SCAND2 RNA was correlated with better prognoses of lung adenocarcinoma and sarcoma. These data suggest that the stress-inducible SCAN-TFs can function as a feedback system, suppressing excessive stress response and inhibiting cancers.

## 1. Introduction

The cell stress response is an intrinsic system in all cells responding and adapting to environmental stimulations. One of the representative stress response systems is the heat shock factor (HSF)–heat shock protein (HSP) program that maintains proteostasis in cells [1,2,3,4] and promotes cancer progression [5,6,7]. The HSF–HSP system was originally found to be activated in response to heat shock stress (HSS) but was subsequently shown to also be induced by oxidative stress, heavy metals, toxins, bacterial infections, and other stresses [1]. Such proteotoxic stresses cause protein misfolding and thus activate the HSF–HSP system. Of note, the HSF–HSP system is often activated in cancer [8,9,10].

Heat shock protein 90 (HSP90) members are stress-inducible protein chaperones that assist protein folding and re-folding to give their clients functionality in the intracellular space. As HSP90 has several hundred protein substrates (called ‘clients’), it is involved in many cellular processes beyond protein folding, which include DNA repair, development, the immune response, and neurodegeneration [11,12,13,14,15]. Elevated expression of HSP90 has been observed in many cancer types and correlates with poor prognosis, increased metastatic potential, and resistance to therapy [16,17,18,19]. Moreover, the HSP90 alpha and beta isoforms are often released with extracellular vesicles (EV), including exosomes, by cancer cells and trigger cancer initiation and progression, as well as the polarization of tumor-associated macrophages (TAM) to an immunosuppressive M2 subtype [6,17,20,21,22]. In addition, HSP90 is produced and released by immunocytes, such as macrophages, and plays a key role in antigen cross-presentation [14,15,23,24]. HSF1 is the master regulator of the protein quality control machinery in response to proteotoxic stress conditions [2,3,25] and enhances cancer progression [5,7]. Upon proteotoxic stress, HSF1 binds to heat shock elements (HSE) in the promoter regions of HSP genes and other stress-inducible genes [2,3,25]. HSF1 drives oncogenesis in many ways beyond inducing the gene expression of chaperones [7,26,27,28], co-chaperones [6], and non-chaperone target genes [9]. However, less is known about how HSP90 genes are attenuated by alternative transcription factors.

The SCAN domain-containing transcription factors (SCAN-TF) contain the SREZBP-CTfin51-AW1-Number 18 cDNA domain (SCAND), a leucine-rich oligomerization domain highly conserved among the SCAN-TF family (Figure S1). This family contains more than 50 members, most of which contain a zinc finger (ZF) domain to scan DNA sequences for binding; hence, they are called SCAN-ZF factors [29,30,31,32,33]. Myeloid zinc finger 1 (MZF1), also known as ZSCAN6 or ZNF42, is a prototypical SCAN-ZF that contains an N-terminal SCAN domain, a linker region, and a C-terminal DNA binding domain [34,35,36]. Many studies have identified MZF1 as an oncogenic transcription factor [34,37,38,39,40] and cancer stemness factor [41,42]. However, depending on the context, MZF1 can also function as a tumor suppressor [43,44,45]. While there are more than 50 types of SCAN-TFs, only 6 zinc-fingerless SCAND-only proteins exist [30,31]. SCAND1 is a SCAN domain-only protein that can hetero-oligomerize with other SCAN-ZFs, including MZF1, through inter-SCAN domain interactions to repress transcription [32,33,37,43,46]. Thus, hetero-oligomerization between SCAND molecules and SCAN-ZF molecules can transform their roles, forming a transcriptional repressor complex [32,33,37,43,46]. Indeed, SCAND1 represses the co-chaperone *CDC37* gene (encoding cell division control 37) by interacting with MZF1 and suppressing prostate cancer [37]. Moreover, SCAND1 and MZF1 are mutually inducible and form oligomers that can reverse epithelial-to-mesenchymal transition (EMT), tumor growth, and migration by repressing EMT driver genes and mitogenic protein kinase (MAPK) genes [43]. High expression of MZF1 correlated with poor prognoses in prostate cancer and kidney cancer, whereas *SCAND1* and *MZF1* expression correlate with better prognoses in pancreatic cancer and stage III head and neck cancers [43]. These suggest that MZF1 alone is oncogenic, whereas repressing complexes of SCAND1 and MZF1 is tumor suppressor, depending on their gene expression in cancer cases. SCAND2 is another member of SCAND factors with high homologies. Of note, SCAND2 RNA has been registered as *SCAND2P*, a pseudogene for long noncoding RNA (lncRNA), and protein-coding SCAND2 mRNA in the NCBI database, although it has not been biologically investigated.

It has been unclear whether the SCAND factors and MZF1 are involved in proteotoxic stress response in cancer. Here, we show that the SCANDs and MZF1 are stress-inducible factors and can attenuate HSP90 gene expression in prostate cancer cells. We also show that cell stress alters the transcript variants of protein-coding and noncoding RNA of SCAND2. Moreover, we show that high expression levels of these SCAN-TF RNA can be predictive biomarkers of better prognoses in several cancer types, indicating potential tumor suppressor roles.

## 2. Results

### 2.1. Heat Shock Elements (HSE) in the Promoter Regions of MZF1(ZSCAN6), SCAND1, and SCAND2P Genes in the Human Genome

**Figure 1:**
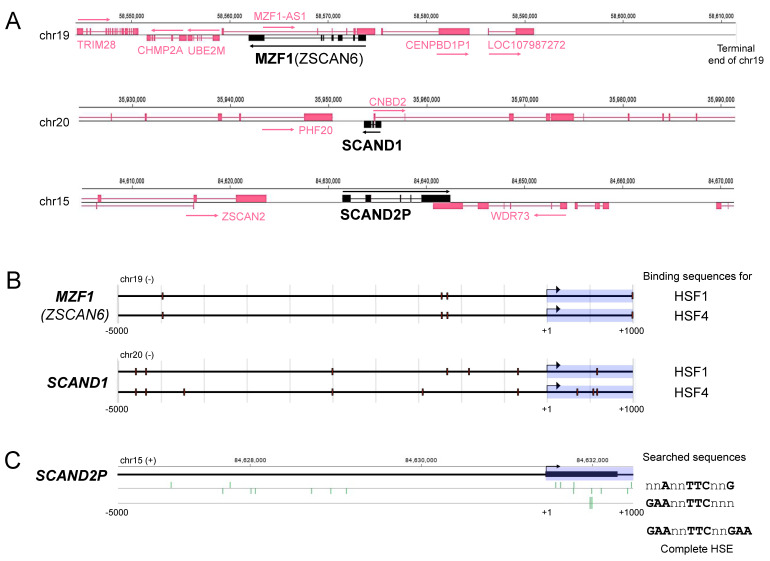
Gentic loci of *MZF1(ZSCAN6), SCAND1*, and *SCAND2P* in the human genome. (**A**) Locations and structures of *MZF1, SCAND1*, and *SCAND2P* genes on human chromosomes (hg38). (**B**,**C**) Mapping of heat shock elements (HSEs) in the promoter regions (−5000 to +1000) of (**B**) *MZF1*, *SCAND1*, and (**C**) *SCAND2P* genes.

We first grasp the loci and structures of *MZF1(ZSCAN6), SCAND1*, and *SCAND2P* genes in the human genome. The MZF1 gene is located at the terminal end of chromosome 19 (Figure 1A, Figures S1 and S2). *SCAND1* gene is located on chromosome 20. *SCAND2P* gene is located on chromosome 15. These SCAN-TF genes overlap with other genes encoding *MZF1-AS1* (antisense 1), *CNBD2*, and *WDR73*. *SCAND2P* gene is located neighboring with *ZSCAN2* gene, another member of SCAN-ZFs. *MZF1* gene is located near the *TRIM28* gene, encoding a stress-related transcriptional elongation factor.

To predict whether *MZF1, SCAND1*, and *SCAND2P* genes are transcriptionally regulated by HSFs and MZF1, we searched for binding sequences of these TFs in promoter regions. Several binding sequences (BSs) for HSF1 and HSF4 were found in the promoter regions of *MZF1, SCAND1*, and *SCAND2P* genes (Figure 1B,C; Table 1). Moreover, dozens of MZF1-BSs were found in the promoter regions of *SCAND1* and *MZF1* genes (Table 1). These data suggested that *MZF1, SCAND1*, and *SCAND2P* genes are transcriptionally regulated by HSFs, MZF1, and SCANDs.

### 2.2. Heat Shock Stress Induces MZF1, SCAND1, and SCAND2 Gene Expression and Reduces lncRNA-SCAND2P in Prostate Cancer

**Figure 2:**
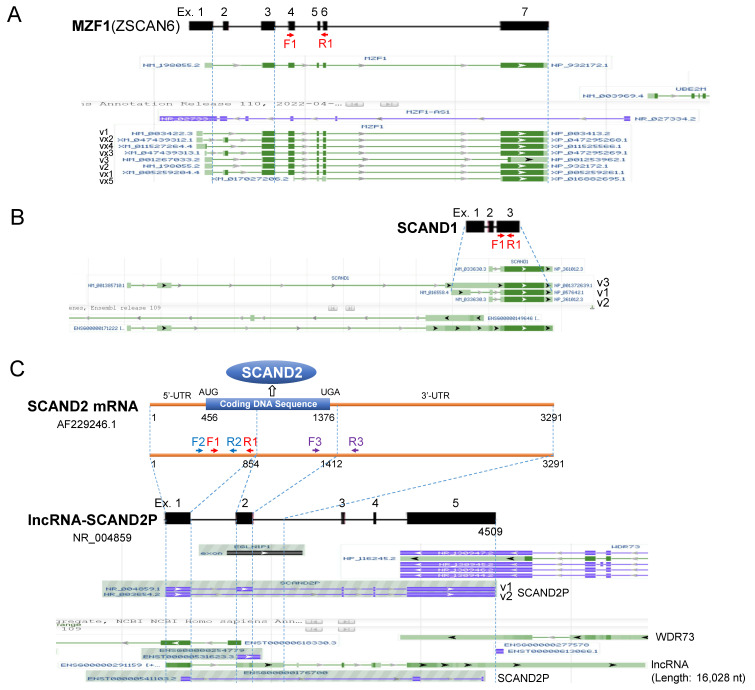
Alternative splicing variants and primer mapping of (**A**) *MZF1(ZSCAN6)*, (**B**) *SCAND1*, and (**C**) *SCAND2* genes. Complete coding DNA sequence (CDS) of SCAND2 mRNA and lncRNA-SCAND2 overlapped in the genome. Ex., exon numbers. Primer positions were mapped, e.g., F1 and R1.

We next considered transcript variants of *MZF1, SCAND1*, and *SCAND2*. We found eight MZF1 RNA variants, three SCAND1 RNA variants, and three SCAND2(SCAND2P) RNA variants in the NCBI database (Figure 2A–C). Of note, the SCAND2 complete coding DNA sequence (AF229246.1) and lncRNA-SCAND2P (NR_004859.1 and NR_003654.2) were found at the same genome locus (Figure 2C). We designed primer pairs that can detect all these variants for qRT-PCR analysis (Figure 2 and Figure S2, Table S1).

**Figure 3:**
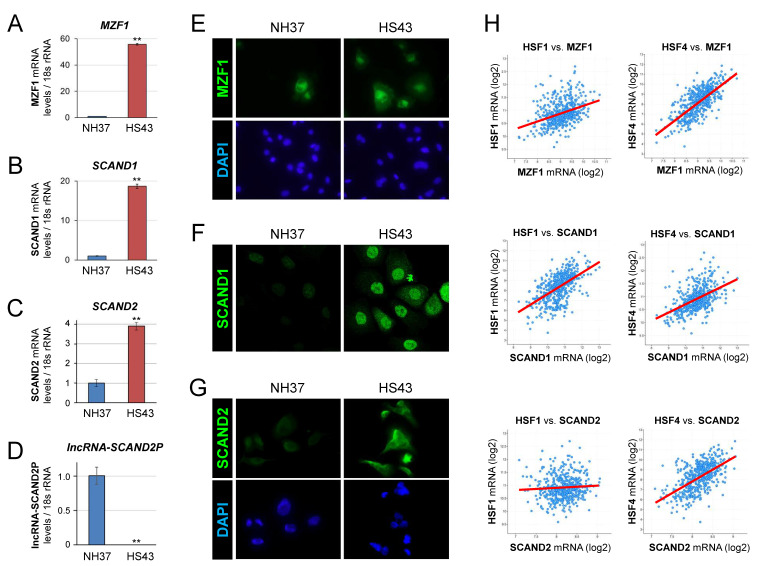
Heat shock stress induces the expression of MZF1(ZSCAN6), SCAND1, and SCAND2 but eliminates lncRNA-SCAND2P in DU-145 prostate cancer cells. (**A**–**D**) qRT-PCR analysis for MZF1 (**A**), SCAND1 (**B**), and SCAND2 (**C**) RNA and lncRNA-SCAND2P (**D**) upon HSS. LncRNA-SCAND2P was detected under the NH37 condition but lost after HSS. NH37, non-heated at 37 °C. HS43, heat-shocked at 43 °C for 30 min. ** *p* < 0.01, n = 3. (**E**–**G**) immunocytochemistry of MZF1 (**E**), SCAND1 (**F**), and SCAND2 (**G**) expressed with or without heat shock. (**H**) Co-expression correlation between *HSF1* or *HSF4* vs. *MZF1*, *SCAND1*, and *SCAND2* in patient-derived prostate adenocarcinoma (494 samples, TCGA PanCancer Atlas).

We then asked whether MZF1, SCAND1, and SCAND2 mRNA expression was inducible by heat shock stress (HSS). MZF1, SCAND1, and SCAND2 mRNA expression was significantly induced by HSS in DU-145 cells (Figure 3A–C and Figure S3). On the other hand, the expression level of lncRNA-SCAND2P was significantly reduced upon HSS (Figure 3D and Figure S3). These data suggested that the alternative transcript balance was shifted from lncRNA-SCAND2P to protein-coding SCAND2 mRNA upon cell stress, which potentially regulates alternative splicing.

To examine the stress inducibility of the SCAN-TFs, we next performed immunocytochemistry after HSS. MZF1, SCAND1, SCAND2 expression levels were increased upon HSS in DU-145 cells (Figure 3E–G and Figure S4). We next examined whether the gene expression of SCAN-TFs (*MZF1, SCAND1*, and *SCAND2(P)*) was correlated with *HSF1* or *HSF4* gene expression in prostate cancer specimens derived from patients. *SCAND1* and *MZF1* gene expression levels were correlated with the degree of gene expression of *HSF1* and *HSF4* (Figure 3H, Table 2). *SCAND2* expression was correlated with the expression of *HSF4* but not with *HSF1*.

These data suggested that these SCAND-TF genes (MZF1, SCAND1, and SCAND2) are highly responsive stress-inducible genes in prostate cancer, whose regulation is intricately mediated by the coordinated action of HSF1 and/or HSF4. Furthermore, cell stress in prostate cancer changes the variant expression of the SCAND2 gene from the lncRNA-SCAND2P to protein-coding SCAND2 mRNA.

### 2.3. Co-Expression Correlation of SCAN-TF Genes in Prostate Cancer

**Figure 4:**
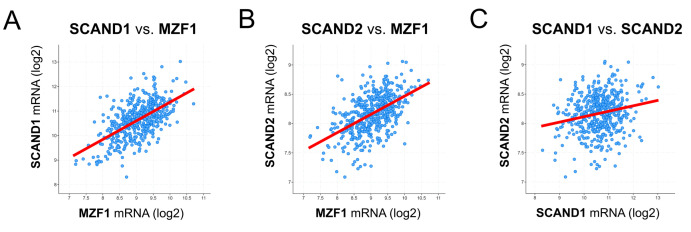
Co-expression correlation between *MZF1*, *SCAND1*, and *SCAND2* in prostate cancer. (**A**) SCAND1 vs. MZF1, (**B**) SCAND2 vs. MZF1, (**C**) SCAND1 vs. SCAND2 in prostate adenocarcinoma specimens derived from patients (494 samples, TCGA PanCancer Atlas).

We recently showed that *MZF1* and *SCAND1* gene expression could mutually induce each other’s expression [43]. Moreover, several MZF1-BSs exist in the promoter regions of *SCAND1* and *MZF1* genes, as shown in Table 2. We analyzed co-expression correlations of *MZF1*, *SCAND1*, and *SCAND2* genes in prostate cancer. In prostate adenocarcinoma specimens, the expression of *MZF1* was positively correlated with both *SCAND1* and *SCAND2* RNA expression (Figure 4A–C; Table 3).

Therefore, these data suggested that MZF1 could induce SCAND1 and SCAND2 gene expression in prostate cancer.

### 2.4. Heat Shock Stress Induces HSF1 and MZF1(ZSCAN6) Binding to HSP90 Genes

**Figure 5:**
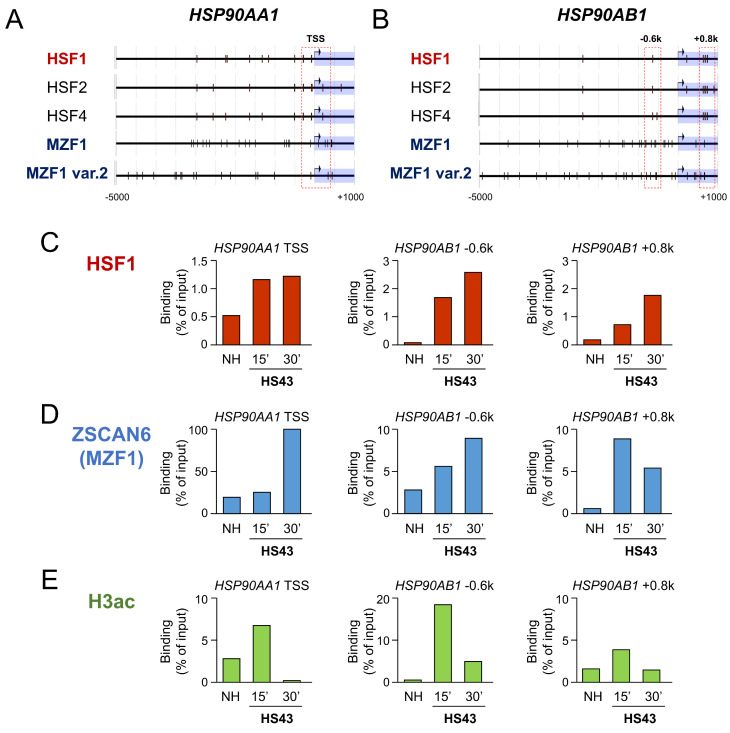
Heat shock stress induces HSF1 and MZF1(ZSCAN6) binding to *HSP90* genes. (**A**,**B**) Promoter regions (−5000 to +1000) of the *HSP90AA1* gene (**A**) and the *HSP90AB1* gene (**B**) mapped with binding sites of HSFs and MZF1(ZSCAN6). Black vertical bars indicate binding sites. Hatched boxes indicate regions analyzed by ChIP-qPCR. (**C**–**E**) ChIP-qPCR assay. PC-3 cells were treated with heat shock at 43 °C (HS43) for 15 or 30 min or non-heated (NH), and chromatin was fixed. ChIP was performed using antibodies against HSF1 (**C**), MZF1/ZSCAN6 (**D**) and acetylated histone H3 (H3ac) (**E**) for qPCR of *HSP90* genes.

To ask whether *HSP90* genes were directly regulated by HSF1 and MZF1/ZSCAN6, we next analyzed promoter regions of *HSP90AA1* and *HSP90AB1* genes and performed ChIP-qPCR. The *HSP90AA1* promoter region (−5000 to +1000) contained 9 sites for HSF1 and 40 binding sites for MZF1. The *HSP90AB1* promoter region (−5000 to +1000) contained 8 binding sites for HSF1 and 50 binding sites for MZF1 (Table 4; Figure 5A,B). These data indicated that *HSP90* genes could be potential targets for the HSF1 and MZF1-SCAND complex.

We next performed ChIP-qPCR analysis to ask about the direct regulation of *HSP90* genes by HSF1 and MZF1(ZSCAN6). The binding of HSF1 to the *HSP90AA1* gene promoter region was increased in response to HSS in PC-3 prostate cancer cells (Figure 5C). MZF1(ZSCAN6) binding to the *HSP90AB1* gene promoter region was also increased in response to HSS (Figure 5D). Histone H3 acetylation, a marker of transcriptional activation, in the *HSP90AA1* gene promoter region was transiently increased in response to HSS in 15 min and then reduced in 30 min (Figure 5E).

These data suggested that HSF1 and MZF1 binding to the *HSP90* gene promoter regions could be transiently activating *HSP90* genes and repressing them later. Induction of SCAND expression upon HSS and its binding to MZF1 may function to turn off the transcription of *HSP90* genes in 30 min.

### 2.5. MZF1 and SCAND1 Blocks the Heat Shock Response of HSP90

**Figure 6:**
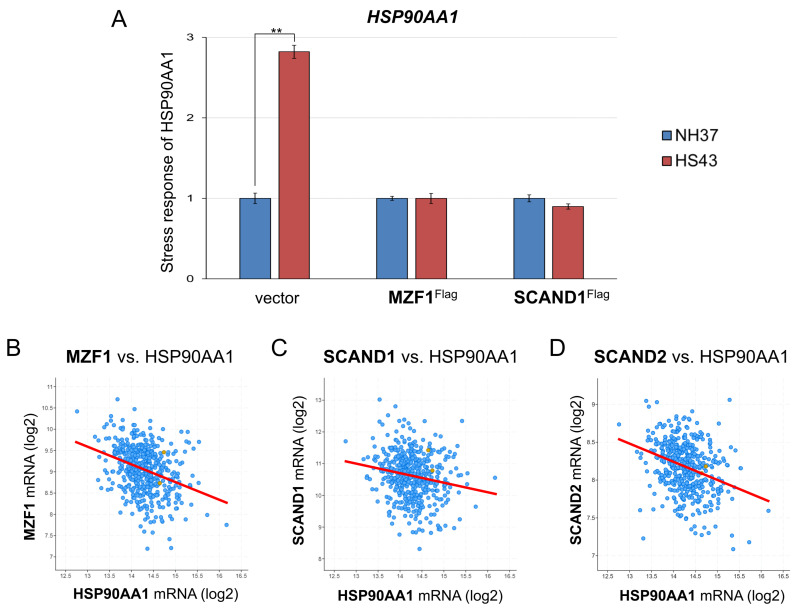
MZF1 and SCAND1 blocked the heat shock response of the *HSP90AA1* gene. (**A**) qRT-PCR for *HSP90AA1* gene. Stable DU-145 cells transfected with pcDNA3.1 vector, pcDNA/MZF1-Flag, and pCMV/SCAND1-Flag were treated with or without HSS for 30 min. ** *p* < 0.01, n = 3. (**B**–**D**) Co-expression correlation between HSP90AA1 vs. MZF1(ZSCAN6) (**B**), SCAND1 (**C**) and SCAND2 (**D**) genes in prostate adenocarcinoma (494 samples, TCGA PanCancer Atlas).

We next examined whether MZF1 and SCAND1 could affect the heat shock response of the *HSP90AA1* gene. Indeed, HSP90AA1 mRNA expression was induced by HSS. However, MZF1 or SCAND1 overexpression blocked the HS response of HSP90AA1 gene expression (Figure 6A).

To ask whether *HSP90AA1* gene was regulated by transcription factors, including SCAN-TFs and HSFs, we examined their co-expression correlation in prostate adenocarcinoma specimens. *HSP90AA1* gene expression was negatively correlated with the gene expression of *MZF1(ZSCAN6), SCAND1, SCAND2*, and *HSF4* in prostate adenocarcinoma specimens (Figure 6B–D; Table 5). *HSP90AA1* gene expression was not significantly correlated with the gene expression of *HSF1, HSF2*, and *HSF5* in prostate adenocarcinoma specimens (Table 5).

We next examined whether the expression of other *HSP* genes is correlated with expression of these SCAN-TFs in addition to *HSP90AA1*. *MZF1, SCAND1*, and *SCAND2* expressions were negatively correlated with gene expression of *HSPA13, HSPA4, HSPA4L,* and *HSPH1* (Table 6 and Table S2). These data suggested that SCAN-TFs co-repressed multiple *HSP* genes.

These data indicated that *HSP90AA1* gene expression was negatively regulated by MZF1(ZSCAN6), SCAND1, SCAND2, and HSF4 in prostate adenocarcinomas.

### 2.6. Reduced Expression of SCAND2 and MZF1 Coincide with the Increased HSP90 Expression in Tumor Tissues Compared with Normal Tissues

**Figure 7:**
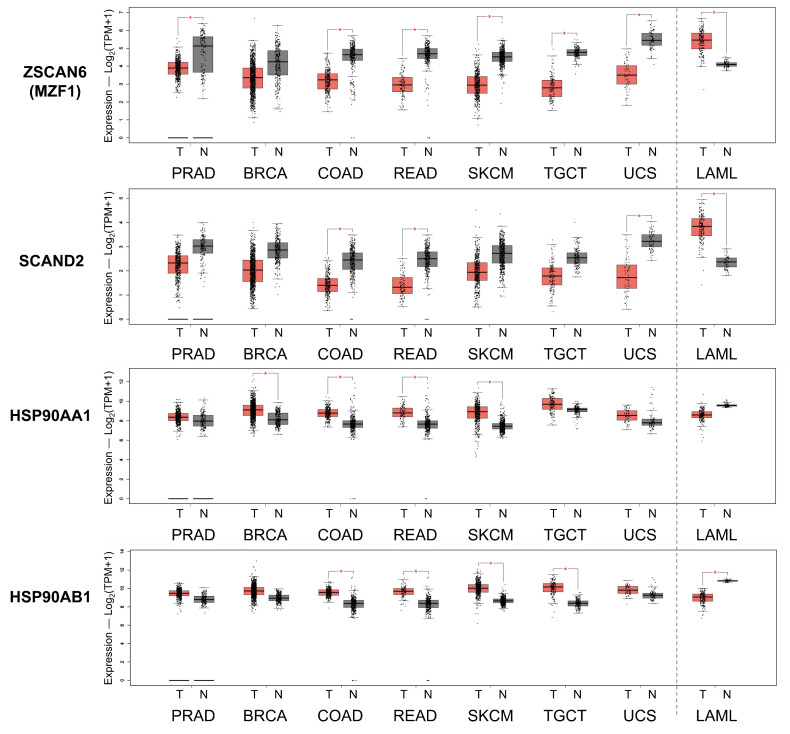
Gene expression levels of *ZSCAN6(MZF1), SCAND2*, and *HSP90* in various tumor types vs. paired normal tissues. Red box, tumor tissues (T). Gray box, normal tissues (N). Prostate adenocarcinoma (PRAD), breast invasive carcinoma (BRCA), colon adenocarcinoma (COAD), rectum adenocarcinoma (READ), skin cutaneous melanoma (SKCM), testicular germ cell tumors (TGCT), uterine carcinosarcoma (UCS), and acute myelo1id leukemia (LAML). Patient-derived clinical samples from TCGA PanCancer Atlas and GTEx were analyzed using GEPIA2. * *p* < 0.01.

We next hypothesized that the repressing factors SCAND2 and MZF1 were reduced in tumor tissues while *HSP90* gene expression was increased in tumor tissues. *SCAND2* and *MZF1(ZSCAN6)* gene expression was lower in prostate adenocarcinoma (PRAD), breast invasive carcinoma (BRCA), colon adenocarcinoma (COAD), rectum adenocarcinoma (READ), skin cutaneous melanoma (SKCM), testicular germ cell tumors (TGCT) and uterine carcinosarcoma (UCS), while *HSP90AA1* and *HSP90AB1* gene expression was higher in these cancer types compared with paired normal tissues (Figure 7 and Figure S5). Exceptionally, *SCAND2* and *MZF1(ZSCAN6)* gene expression was higher in acute myeloid leukemia (LAML), while the expression levels of *HSP90AA1* and *HSP90AB1* genes were lower, compared with paired normal tissues.

These data suggested that reduced expression of SCAND2 and MZF1 could result in the elevated expression of *HSP90* genes in tumor tissues in many cancer types.

### 2.7. SCANDs and MZF1(ZSACAN6) Expression Correlates with Enhanced Prognoses Whereas HSP90 Expression Is Correlated with Poor Prognoses in Cancers

**Figure 8:**
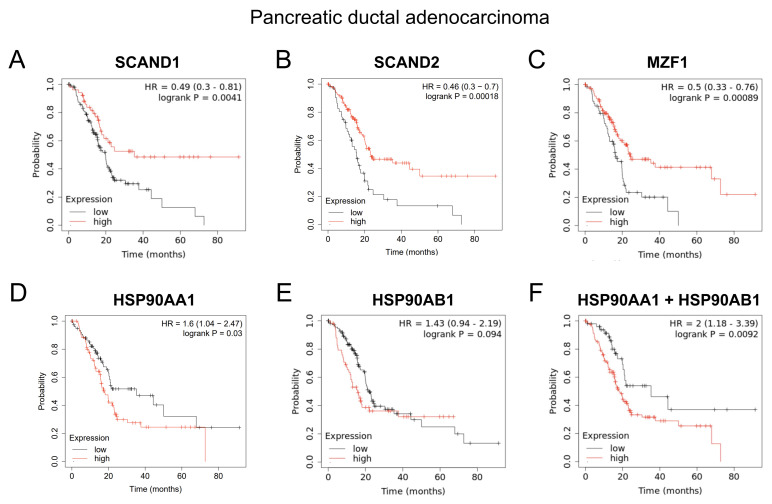
Kaplan–Meier plots showing prognostic values of *SCANDs, MZF1*, and *HSP90* gene expression in pancreatic cancer. Data were from TCGA PanCancer Atlas, pancreatic adenocarcinoma (PAAD), n = 177. High expression of *SCANDs* and *MZF1* genes (**A**–**C**) are correlated with better prognoses whereas high expression of *HSP90* genes (**D**–**F**) are correlated with poor prognosis of pancreatic DAC. Data in panels A and C were published in: Eguchi, T., et al., 2022 [43].

We next hypothesized that the repressive transcription factors SCAND1, SCAND2 and MZF1(ZSCAN6) would contribute to enhanced prognosis in cancer patients, whereas high expression of *HSP90* genes would likely be involved in a poorer prognosis. High expression levels of *SCAND1, SCAND2* and *MZF1* genes were significantly correlated with enhanced prognosis of patients suffering from pancreatic ductal adenocarcinoma (DAC) (Figure 8A–C; Table 7). High expression levels of *HSP90AA1* and *HSP90AB1* (associated with a low *MZF1* expression scenario) were significantly correlated with poorer prognosis of patients suffering from pancreatic cancer (Figure 8D–F).

**Figure 9:**
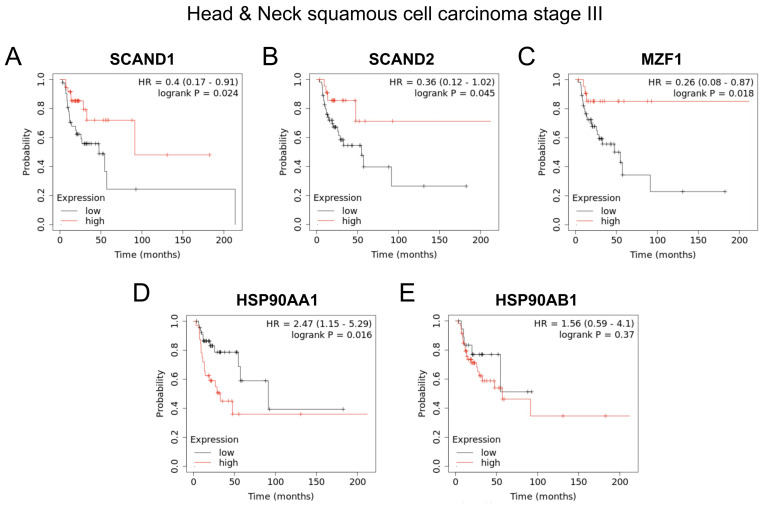
Kaplan–Meier plots showing prognostic values of *SCANDs, MZF1,* and *HSP90* gene expression in head and neck cancers. Data were from TCGA PanCancer Atlas, head and neck squamous cell carcinoma (HNSC) (stage III), n = 78. High expressions of *SCANDs* and *MZF1* (**A**–**C**) are correlated with better prognosis, whereas high expressions of *HSP90* (**D**,**E**) are correlated with poor prognosis of head and neck SCC. Data in panels A and C were published in: Eguchi, T., et al., 2022 [43].

Similarly, high expressions of *SCAND2, SCAND1*, and *MZF1* genes were significantly correlated with enhanced prognosis of patients suffering from stage III head and neck squamous cell carcinoma (SCC) (Figure 9A–C), whereas high expression levels of *HSP90AA1* and *HSP90AB1* genes were significantly correlated with poorer prognosis of patients suffering from stage III head and neck SCC (Figure 9D,E).

High expression of SCAND2 and MZF1 was significantly correlated with enhanced prognosis of patients suffering from lung adenocarcinoma (Figure S6A–C). Consistent with this, high expression of *HSP90AA1* and *HSP90AB1* was significantly correlated with poorer prognosis of patients suffering from lung adenocarcinoma (Figure S6D,E).

Moreover, high expression of *SCAND2* was correlated with enhanced prognoses in sarcoma and cervical SCC (Table 7).

These data suggested that high expression of *SCAND2*, *SCAND1*, and *MZF1* genes were superior prognostic markers in several cancer types, including pancreatic cancer, head and neck cancers, lung adenocarcinoma, sarcoma and cervical cancer.

## 3. Discussion

**Figure 10:**
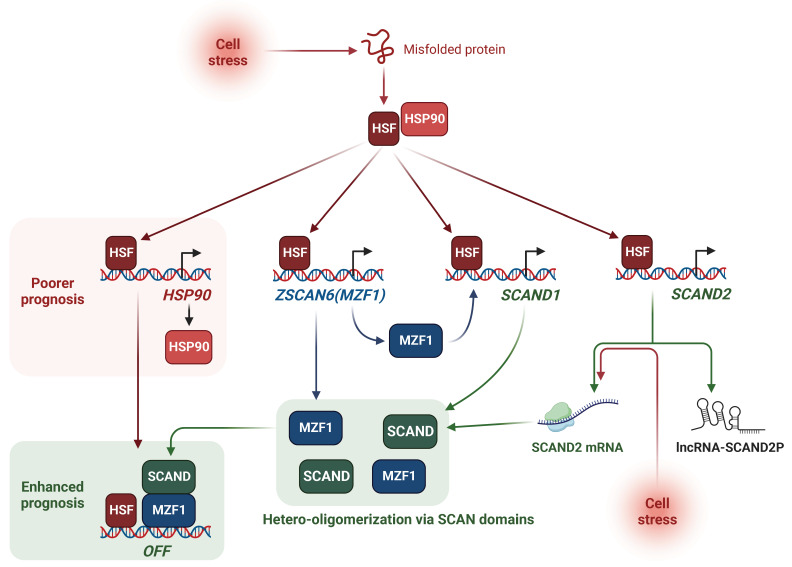
Stress-inducible SCAN transcription factors SCAND and MZF1 repress *HSP90* gene expression. Cell stress activates HSF1 that binds to HSEs in the promoter regions of the *HSP90* genes (*HSP90AA1* and *HSP90AB1*), *ZSCAN6(MZF1)* gene, and *SCAND* genes (*SCAND1* and *SCAND2*). MZF1 induces SCAND1 expression. Cell stress also switches the SCAND2 transcript variants from lncRNA-SCAND2P to SCAND2 mRNA. Hetero-oligomers of MZF1 and SCAND bind to and repress *HSP90* genes. High expression of HSP90 is a biomarker of poorer prognosis, while SCAN-TF complexes repress the transcription of *HSP90* gene and enhance the prognosis of cancer patients.

We have shown that the cell stress-inducible SCAND1 and MZF1 genes repress the stress response of the HSF–HSP system (Figure 1, Figure 2, Figure 3, Figure 4, Figure 5 and Figure 6). SCAND1, SCAND2, and MZF1/ ZSCAN6 are heat-inducible and could form repressing complexes on *HSP90* genes (Figure 10) [37,43]. These findings were consistent with the data from clinical tumor specimens. SCAND2 and MZF1 RNA were expressed at higher levels in normal tissues than in paired tumor tissues (Figure 7). In contrast, HSP90 RNA was expressed at higher levels in tumor tissues than in paired normal tissues in many cancer types (Figure 7). These data suggest that SCAND2/MZF1 hetero-oligomers could inhibit the excess stress response of HSP90 expression in normal tissues, whereas loss of expression of these SCAN-TFs could result in the gain of HSP90 in tumor tissues. We showed that high expression of SCAN-TFs (SCAND1, SCAND2, and MZF1) were predictive biomarkers of enhanced prognoses for patients suffering from pancreatic cancer and head and neck cancers (Figure 8 and Figure 9). Moreover, high expression of SCAND2 (and/or lncRNA-SCAND2P) was a predictive biomarker of enhanced prognoses for patients suffering from lung adenocarcinoma, sarcoma, and cervical cancer (Table 7, Figure S6). These data indicate that SCAND/MZF1 repressing complexes are potentially tumor suppressing, contributing to better prognoses of patients suffering from several cancer types.

Our data, for the first time, indicate that SCAND2 RNA expression is a novel biomarker of better prognoses in cancer patients (Table 7, Figure 8 and Figure 9). Only one group has previously reported the existence of the *SCAND2* gene [47]. Moreover, SCAND2 has been registered as *SCAND2P*, a pseudogene for lncRNA (Ref seq ID: NR_004859.1 and NR_003654.2). Gene expression data of *SCAND2* (or *SCAND2P*) were found in many databases. Of note, the protein structure of SCAND2 found in Phosphosite plus is more conserved with the N-terminal region of MZF1(ZSCAN6) than SCAND1 (Figure S1). Moreover, complete coding DNA sequences of SCAND2 mRNA are found in the NCBI database (GenBank ID: AF229246.1 (coding 306 aa, AAG33966.1), AK022844.1 (coding 152 aa, BAB14268.1), and AK290489.1 (coding 152 aa, BAF83178.1)) (Figure 2 and Figure S2). Our research highlights that HSS can shift the transcript balance from the lncRNA to the protein-coding mRNA of SCAND2 (Figure 3 and Figure S3), potentially via changing alternative splicing. SCAND2 and MZF1 RNA were each expressed in normal tissue at higher levels than in tumor tissues (Figure 7), whereas SCAND1 RNA expression did not show this pattern. Therefore, SCAND2 may form more stable hetero-oligomers with MZF1(ZSCAN6) than SCAND1 to repress oncogenic gene expression in tumors. Further functional analysis of SCAND2 is required for this novel gene.

Our data also suggested that cell stress regulates RNA variants of the *SCAND2* gene, potentially via alternative RNA splicing, alternative RNA polyadenylation, and/or protein translational control (Figure 2, Figure 3 and Figures S2–S4). Cell stresses, including oxidative stress and cancer therapy-induced stress, have been reported to regulate alternative RNA splicing via the Hu antigen R (HuR), also known as ElavL1 [9,48,49]. We have reported that HSF1 regulates β-catenin RNA, which contains many AU-rich sequences, in mammary cancer cells by controlling HuR/ElavL1 expression [9]. The Hu/Elav RNA-binding protein family, composed of HuR (also known as HuA), HuB, HuC, and HuD), regulate alternative splicing [8,50,51,52], while HuR is the most investigated member that binds to AU-rich sequences of RNA. Cell stress also modulates the function of the splicing regulatory protein RBM4 in translation control [53]. Therefore, alternative expression of SCAND2 RNA variants, including lncRNA-SCAND2P and SCAND2 mRNA, could be regulated by Hu and/or RBM4 RNA-binding proteins under cell-stressed conditions.

Our study also revealed a striking correlation between the expression of HSF1 and the SCAN-TF genes (SCAND1 and MZF1) (Figure 3). Furthermore, we have identified HSF4 as a potential inducer of SCAN-TF gene expression, including SCAND1, SCAND2 and MZF1. HSF4 lacks a leucine zipper 4 (LZ4) domain, resulting in its constitutive trimerization and DNA-binding activity [54]. Several HSF4-BSs were found in the promoter regions of *SCAND1, SCAND2*, and *MZF1* genes (Table 1). Thus, the expression of HSF4 could result in the constitutive expression of *SCAND2, SCAND1*, and *MZF1* without requiring cellular stress. While HSF4 is known to be oncogenic in several cancer types, such as colorectal cancer, hepatocellular carcinoma, and lymphoma [55,56,57], our data suggested that HSF-dependent expression of SCAN-TFs could actually reduce oncogenic gene expression in tumors.

Moreover, our data suggested that the stress-inducible SCAND–MZF1 complex represses the *HSP90AA1* gene while also repressing other HSPs and many more stress-responsive genes (Table 6 and Table S2). We have recently shown SCAND1 and MZF1 expression to negatively correlate with EMT driver genes, including *ZEB1, CTNNB1* and *TGFBR1/2/3*, and mitogenic genes encoding kinases in the MEKK–MEK–ERK signaling pathway [43]. Moreover, SCAN-only family genes and MZF1 expression were negatively correlated with the expression of NF-κB signaling molecules and PI3K-AKT signaling molecules. Thus, we have shown that EMT, some oncogenic signaling pathways, and the HSF–HSP gene expression system are all key targets of the SCAND–MZF1 repression complexes.

Our data also suggested that tumors’ stress levels differs among clinical cases (Figure 7). Tumor cells are characteristically exposed to various stresses from the microenvironment, such as immune/inflammatory stress [19], therapeutics [18], hypoxia [22,58,59,60,61], acidification [62,63], hyperthermia [64,65] or heat stress [4,6,19,25,28], endoplasmic reticulum stress [66], nuclear envelope stress [67,68], replication stress [69], oxidative stress [70], mechanical stress, osmotic stress, and genotoxic (DNA damage) [71,72] and proteotoxic stress [1,2,4,73]. Therefore, it might be difficult to determine the types and levels of stresses in each tumor. However, there were strong correlations between the RNA expression of SCAN-TFs and HSP90AA1 in clinical tumor specimens (Figure 6, Figure 7, Figure 8 and Figure 9). These clinical data support the hypothesis that the SCAN-TF complexes repress excessive HSP gene expression and suppress tumors.

In conclusion, we have demonstrated that the cell stress-inducible SCAND and MZF1 repress the stress response in cancer. MZF1 and SCAND1 are mutually inducible and can form a repressive complex on the *HSP90* gene promoters. Moreover, cell stress changed the transcript variants from the lncRNA-SCAND2P into protein-coding SCAND2 mRNA. Nevertheless, elevated levels of SCAND2 RNA are novel potential markers of better prognoses in several cancer types, including pancreatic cancer, head and neck cancers, lung adenocarcinoma, sarcoma, and cervical cancer. This effect may ensue from the findings that the SCAND–MZF1 repressive system is important for preventing cancer-related gene expression physiologically while playing a key role in tumor suppression.

## 4. Materials and Methods

### 4.1. Cell Culture and Heat Shock Stress

Prostate cancer cell lines DU-145 and PC-3 were provided by ATCC and cultured in DMEM and RPMI medium, respectively, with 10% FBS. For HSS, the medium was replaced with pre-warmed medium at 43 or 37 °C and then put in a water bath at 43 or 37 °C, as described previously [6,28].

### 4.2. Genome and Promoter Analysis

We used the Subio platform (subioplatform.com, accessed on 20 February 2023) for genome analysis. The human genome (hg38, GRCh38.p14) sequence was downloaded from the UCSC server (hgdownload.soe.ucsc.edu/goldenPath/hg38/chromosomes, accessed on 20 February 2023). We used ‘Find Regions from Seq’ plug-in to seek HSEs (5’-nnAnnTTCnnG-3’ and 5’-GAAnnTTCnnn-3’) in SCAND2P gene. We also used the Eukaryotic Promoter Database (EPD) (epd.epfl.ch//index.php, accessed on 20 February 2023) [68]. Promoter IDs (MZF1_1, SCAND1_1, HSP90AA1_1, and HSP90AB1_1) from −5000 to +1000 bp relative to TSS were analyzed with a cut-off *p*-value of 0.001. We searched TF-binding motifs using the Library of Transcription Factor Motifs (JASPAR CORE 2018 vertebrates).

To seek HSEs, we used the Eukaryotic Promoter Database (EPD) with a cutoff value of *p* < 0.001 for MZF1 and SCAND1 genes and the Subio platform for the SCAND2P gene.

### 4.3. qRT-PCR

Primer pairs for RNA of MZF1, SCAND1, SCAND2, and lncRNA-SCAND2P were designed to cover all transcript variants with the assistance of Primer3Plus (Table S1, Figure S2 and Figure 2). The qRT-PCR was performed as previously described [37,74]. To analyze MZF1 and SCAND1 RNA, total RNA was prepared with *DNase* I treatment using RNeasy columns (Qiagen, Hilden, Germany). Synthesis of cDNA was carried out using the QuantiTect kit (Qiagen)and a mixture of oligo dT and random primers, then diluted 5-fold in 10 mM Tris-Cl and 0.1 mM EDTA buffer. A step dilution of the cDNA pool was prepared as a standard for relative expression. Aliquots of cDNA (4–10 µL), 0.25 µM of each primer and 10 µL SYBR green 2× Master Mix (Applied Biosystems, Waltham, MA, USA) were mixed and made up to a 20 µL reaction mixture. The qPCR and melting curve analyses were performed using the StepOnePlus Realtime PCR system (Applied Biosystems, Waltham, MA, USA). To analyze SCAND2 mRNA and lncRNA, total RNA was extracted from cells using TRI Reagent (MRC, Cincinnati, OH). After DNase I treatment, cDNA was synthesized using an iScript cDNA synthesis kit (Bio-Rad, Hercules, CA, USA) and quantified using Micro-Spectophotometer CB2800 (CLUBIO). To perform the qPCR, 10 µL of SsoAdvanced Universal SYBR Green Supermix (2×) (Bio-Rad), 0.25 µM of each primer, and 100 ng of cDNA were mixed and made up to a 20 µL reaction mix volume. The qPCR and melting curve analyses were performed using a CFX96 Realtime PCR system (Bio-Rad).

### 4.4. Plasmids and Transfection

We used pcDNA3.1 (as a control), pcDNA3/MZF1^Flag^, and pCMV6/SCAND1^myc-Flag^ (variant 1, purchased from OriGene, accession number NM_016558) as previously described [37]. DU-145 cells were transfected with these plasmids using Lipofectamine 2000 (Thermo Fisher Scientific, Waltham, MA, USA) and cultured with 0.8 μg/mL geneticin for 2 weeks to establish stable clones as described previously [43].

### 4.5. ChIP

ChIP-qPCR was performed as previously described [37]. Briefly, PC-3 cells were cultured in 150 mm dishes and heat-shocked for 0, 15, and 30 min. ChIP was performed using a magnetic beads-based ChIP assay kit (Merck/Millipore, Kenilworth, NJ, USA). Briefly, endogenous proteins/DNA were cross-linked with 5% formaldehyde. Cells were collected by cell scrapers, centrifuged at 600× *g* for 5 min and resuspended in a ChIP buffer (10 mM Tris, pH 8.0, 200 mM KCl, 1 mM CaCl_2_, 0.5% NP-40) containing a protease phosphatase inhibitor cocktail (Sigma-Aldrich, Burlington, MA, USA). Cells were treated with 3 cycles of sonication on ice with a sonicator. One cycle was a 5 sec sonication with a 15 sec interval at 100% power. OD260 was measured to obtain brief reference DNA concentrations. Sonicated cells containing 10–20 μg DNA or 1 × 10^6^ cells were treated with *MNase* at a final concentration of 100 unit/mL in 500 μL of ChIP buffer and incubated at 37 °C for 40 min, then centrifuged at 15,000× *g* at 4 °C for 10 min. Sheared chromatin DNA in the supernatants was analyzed by 2% agarose gel electrophoresis. For antibody-beads preparation, 20 µL M280 sheep anti-rabbit IgG magnetic beads (Thermo Fisher Scientific) with 2 µg antibodies against MZF1 (C10502, Assay Biotechnology, Fremont, CA, USA), HSF1 (#4356, Cell Signaling Technology, Danvers, MA, USA) or acetylated histone H3 (06-599, Millipore) were mixed in 500 µL ChIP buffer and then rotated for 3 h or overnight. DNA was purified using a QIAquick PCR Purification Kit (Qiagen). Primer pairs for ChIP-qPCR were designed using EPD and Primer3Plus as previously described [37] and listed (Table 8). The qPCR was performed as described above.

### 4.6. Immunocytochemistry and Confocal Laser Scanning Microscopy (CLSM)

Immunocytochemistry and CLSM were performed as previously described [74,75]. Cells were cultured on 12 mm round coverslips coated with poly-D-Lysine/Laminin (BD Bioscience, Franklin Lakes, NJ). Cells were fixed with 4% paraformaldehyde for 20 min, then permeabilized with 0.1% Triton X-100 in PBS for 10 min. Cells were incubated in a blocking buffer containing 1% bovine serum albumin (for MZF1 and SCAND2) or, alternatively, 3% normal goat serum (for SCAND1) in PBS for 30 min, incubated with primary antibodies at 4 °C overnight and then with secondary antibodies at RT for 1 h in the blocking buffer. Cells were washed thrice with PBS for 5 min between the steps. Cells were mounted within ProLong Gold Antifade Mountant (Thermo Fisher Scientific). Fluorescence images were acquired using Axio Vision CLSM (Zeiss, Oberkochen, Germany) with an AxioCam MR3 (Zeiss) camera for SCAND1, and alternatively, FSX100 inverted microscope (Olympus, Tokyo, Japan) for MZF1 and SCAND2. We used antibodies against MZF1 (C10502, Rb pAb, Assay Biotechnology, Fremont, CA), SCAND1 (ab64828, Rb pAb, Abcam), SCAND2 (5F1, H00054581-M02, Ms mAb, Thermo Fisher Scientific), and anti-rabbit IgG conjugated with Alexa Fluor 488 (Thermo Fisher Scientific).

### 4.7. Co-Expression Analysis

A data set of prostate adenocarcinomas (Project ID: TCGA-PRAD, PanCancer Atlas; 494 patients/samples) was analyzed with Spearman’s rank correlation coefficient of co-expression using cBioPortal [76,77].

### 4.8. Gene Expression Profiling of Tumors vs. Paired Normal Tissues

The gene expression profile across tumor samples and paired normal tissues was analyzed using GEPIA2 (gepia2.cancer-pku.cn) to draw box-whisker-scatter plots [78]. Tumor samples from TCGA PanCancer Atlas (gdc.cancer.gov/about-data/publications/pancanatlas, accessed on 20 October 2022) were matched with TCGA normal samples and GTEx data (gtexportal.org, accessed on 20 December 2022) (Table S3) [79]. The *p*-value cutoff was 0.01 as default. Graphs were expressed as a log scale.

### 4.9. Kaplan–Meier Analysis

Kaplan–Meier plotting from RNA-seq data was performed using KM plotter (kmplot.com/analysis, accessed on 20 October 2022) [80]. Data from TCGA PanCancer Atlas were analyzed, including the overall survival of patients suffering from pancreatic ductal adenocarcinoma (n = 177), head and neck squamous cell carcinoma (stage III) (n = 78), lung adenocarcinoma (n = 504), sarcoma (n = 259), and cervical SCC (n = 304) with auto-select best cutoff.

### 4.10. Statistics

Values of two groups were compared with an unpaired Student’s *t*-test. Values of *p* < 0.05 or < 0.01 were considered to indicate statistical significance unless otherwise specified. Data were expressed as Mean ± SD unless otherwise specified.

## Figures and Tables

**Table 1:** The numbers of binding sites for HSF1, HSF4 and MZF1 in the promoter regions of SCAND1 and MZF1 genes.

Gene Promoter ^1^	HSF1-BS	HSF4-BS	MZF1-BS	MZF1-BS var.2
**SCAND1**	7	9	5	15
**MZF1**	4	4	27	30

^1^ Promoter regions from −5000 to +1000 were analyzed. Numbers of binding sequences with *p*-values > 0.001 were counted.

**Table 2:** Co-expression correlation between *HSFs*, *MZF1*, and *SCANDs* in prostate adenocarcinomas.

Gene	Correlated Gene	Spearman’s Correlation ^1^	*p*-Value ^2^	q-Value ^3^
**HSF1 vs.**	**MZF1**	**0.375**	**9.52 × 10^–18^**	**7.37 × 10^–17^**
**HSF1 vs.**	**SCAND1**	**0.518**	**6.73 × 10^–35^**	**2.92 × 10^–33^**
HSF1 vs.	SCAND2	0.0925	4.12 × 10^–2^	6.42 × 10^–2^
**HSF4 vs.**	**MZF1**	**0.705**	**1.97 × 10^–74^**	**3.03 × 10^–72^**
**HSF4 vs.**	**SCAND1**	**0.585**	**3.99 × 10^–46^**	**1.86 × 10^–44^**
**HSF4 vs.**	**SCAND2**	**0.55**	**5.55 × 10^–40^**	**1.89 × 10^–38^**

^1^ Spearman’s correlation > 0.3 were shown in bold. ^2^ *p*-Values < 1 × 10^–15^ were shown in bold. ^3^ q-Values < 1 × 10^–15^ were shown in bold. n = 494.

**Table 3:** Co-expression correlation between *HSFs*, *MZF1*, and *SCANDs* in prostate adenocarcinomas.

Gene	Correlated Gene	Spearman’s Correlation ^1^	*p*-Value ^2^	q-Value ^3^
**MZF1 vs.**	**SCAND1**	**0.548**	**1.27 × 10^–39^**	**6.16 × 10^–38^**
**MZF1 vs.**	**SCAND2**	**0.524**	**1.01 × 10^–35^**	**3.95 × 10^–34^**
SCAND1 vs.	SCAND2	0.170	2.20 × 10^–4^	4.16 × 10^–4^

^1^ Spearman’s correlation > 0.5 were shown in bold. ^2^ *p*-Values < 1 × 10^–30^ were shown in bold. ^3^ q-Values < 1 × 10^–30^ were shown in bold. n = 494.

**Table 4:** The number of binding sites for HSF1, HSF4 and MZF1 in the promoter regions of the *HSP90AA1* and *HSP90AB1* genes.

Promoter	HSF1-BS	HSF4-BS	MZF1-BS	MZF1-BS var.2
**HSP90AA1**	9	12	22	18
**HSP90AB1**	8	8	22	28

Promoter regions from −5000 to +1000 were analyzed. Numbers of binding sequences with *p*-values > 0.001 were counted.

**Table 5:** The negative correlation of gene expression of HSP90AA1 vs. MZF1, SCANDs, and HSFs in prostate adenocarcinoma specimens.

Gene	Correlated Gene	Spearman’s Correlation ^1^	*p*-Value ^2^	q-Value ^3^
HSP90AA1 vs.	**MZF1**	**−0.321**	**3.63 × 10^–13^**	**3.35 × 10^–11^**
HSP90AA1 vs.	**SCAND2**	**−0.32**	**4.34 × 10^–13^**	**3.88 × 10^–11^**
HSP90AA1 vs.	**SCAND1**	**−0.188**	**2.86 × 10^–5^**	**1.71 × 10^–4^**
HSP90AA1 vs.	**HSF4**	**−0.241**	**6.97 × 10^–8^**	**9.73 × 10^–7^**
HSP90AA1 vs.	HSF1	−0.045	3.21 × 10^–1^	4.38 × 10^–1^
HSP90AA1 vs.	HSF2	−0.0164	7.17 × 10^–1^	7.94 × 10^–1^
HSP90AA1 vs.	HSF5	−0.00297	9.48 × 10^–1^	0.963 × 10^–1^

^1^ Spearman’s correlation < –0.15 were shown in bold. ^2^ *p*-Values < 1 × 10^–4^ were shown in bold. ^3^ q-Values < 1 × 10^–3^ were shown in bold. n = 494.

**Table 6:** The negative correlation of HSPs vs. MZF1, SCAND1, and SCAND2 gene expression in prostate adenocarcinoma specimens.

	vs. MZF1		vs. SCAND1		vs. SCAND2	
Correlated Gene	Spearman’s Correlation ^1^	*p*-Value ^2^	Spearman’s Correlation	*p*-Value	Spearman’s Correlation	*p*-Value
**HSPA13**	**−0.448**	**1.75 × 10^–25^**	**−0.572**	**7.79 × 10^–44^**	**−** **0.357**	**4.18 × 10^–16^**
**HSPA4**	**−0.358**	**3.41 × 10^–16^**	**−0.303**	**7.97 × 10^–12^**	**−** **0.394**	**1.52 × 10^–19^**
**HSPA4L**	**−0.326**	**1.58 × 10^–13^**	**−0.411**	**2.66 × 10^–21^**	−0.0341	4.52 × 10^–1^
**HSP90AA1**	**−0.321**	**3.63 × 10^–13^**	−0.188	2.86 × 10^–5^	**−** **0.32**	**4.34 × 10^–13^**
**HSPH1**	**−0.300**	**1.42 × 10^–11^**	−0.191	2.20 × 10^–5^	**−** **0.312**	**1.67 × 10^–12^**

^1^ Spearman’s correlation < –0.25 were shown in bold. ^2^ *p*-Values < 1 × 10^–10^ were shown in bold.

**Table 7:** SCAND1, SCAND2, and MZF1 expression correlate with enhanced prognoses in cancer.

Cancer Type	Log-Rank P			N
	SCAND2	SCAND1	MZF1	
**Pancreatic adenocarcinoma**	0.00018 ***	0.0041 **	0.0009 **	177
**Head & Neck SCC (stage III)**	0.045 *	0.024 *	0.018 *	78
**Lung adenocarcinoma**	0.00032 ***	0.32	0.21	504
**Sarcoma**	0.0096 **	0.11	0.14	259
**Cervical SCC**	0.015 *	0.34	0.21	304

* *p* < 0.05, ** *p* < 0.05, *** *p* < 0.0001.

**Table 8:** Primer sequences for ChIP-qPCR.

Primer Name	Sequences (5′ to 3′)
HSP90AA1 h −100F	GGCTGGGGAGGGTTCTTC
HSP90AA1 h +200R	GAGGCCTCCGGAATAGAAAG
HSP90AB1 h −800F	CCTGAGGATTGGGCTGGTA
HSP90AB1 h −430R	CATCTGCCCTACACATCTCG
HSP90AB1 h +600F	GTCTCCAGCACCCGATACTC
HSP90AB1 h +900R	GAACAGGACCAAACCCAAGA

## Data Availability

Data are available at the Eukaryotic Promoter Database (epd.epfl.ch//index.php, accessed on 20 February 2023), TCGA PanCancer Atlas (gdc.cancer.gov/about-data/publications/pancanatlas, accessed on 20 October 2022), GTEx (gtexportal.org/home/datasets, accessed on 20 February 2023), cBioPortal (cbioportal.org/, accessed on 20 October 2022), GEPIA2 (gepia2.cancer-pku.cn, accessed on 20 December 2022), and KM plotter (kmplot.com, accessed on 20 October 2022).

## Supplementary Materials

The supporting information can be downloaded at https://www.mdpi.com/article/10.3390/ijms24065168/s1.
