# Supplementary material for: Stress-Inducible SCAND Factors Suppress the Stress Response and Are Biomarkers for Enhanced Prognosis in Cancers
Source: Int J Mol Sci. 2023 Mar 8;24(6):5168. doi: 10.3390/ijms24065168 (PMC10049278; doi:10.3390/ijms24065168)
Supplement: Supplementary file 1 [file ijms-24-05168-s001.zip › ijms-2207827-supplementary.pdf]

**Supplementary Materials**

# Stress-inducible SCAND factors suppress stress response and are biomarkers for enhanced prognosis in cancers

**Mona Sheta [1,2], Kunihiro Yoshida [1, 3], Hideka Kanemoto [3], Stuart K. Calderwood [3], and Takanori Eguchi [1,*]**

[1] Department of Dental Pharmacology, Faculty of Medicine, Dentistry and Pharmaceutical Sciences, Okayama University, Okayama 700-8525, Japan
[2] Department of Cancer Biology, National Cancer Institute, Cairo University, Cairo 11796, Egypt
[3] Department of Oral and Maxillofacial Surgery, Graduate School of Medicine, Dentistry and Pharmaceutical Sciences, Okayama University, Okayama 700-8525, Japan
[4] Department of Radiation Oncology, Beth Israel Deaconess Medical Center, Harvard Medical School, Boston, MA 02115, USA

* Correspondence: eguchi@okayama-u.ac.jp; Phone: +81 86 235 6661, Fax +81 86 235 6664

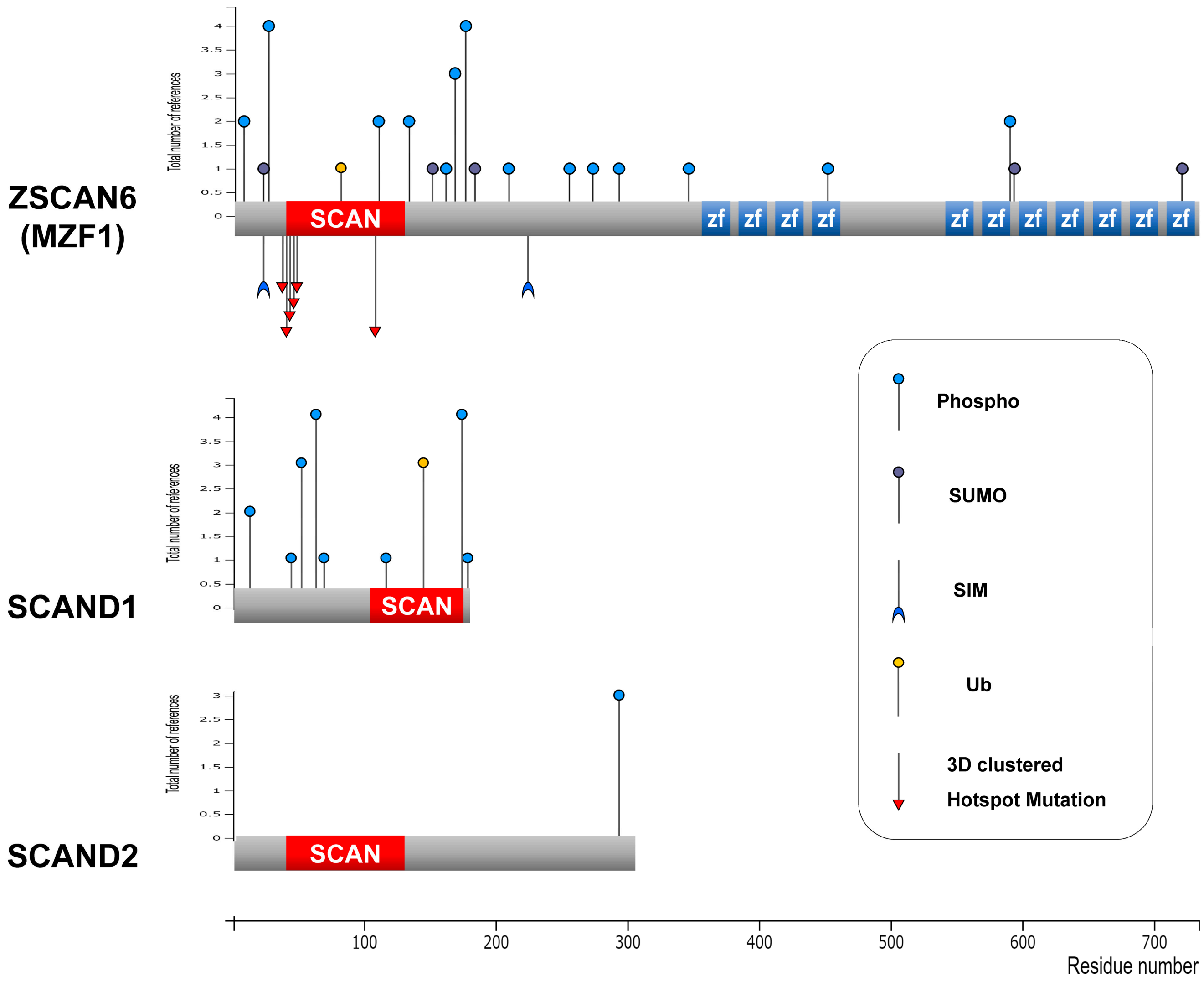


**Figure S1.** Protein structures of MZF1(ZSCAN6), SCAND1, and SCAND2. Post-translational modifications (PTM) and hotspot mutation were mapped. SCAN, SCAN domain. zf, zinc finger domain. Phospho, phosphorylation site. SUMO, sumoylation site. SIM, SUMO interaction motif. Ub, ubiquitination site. Some PTMs were shown in a ref. (Eguchi T et al., 2015, J Cell Biochem 116). This figure was shown as a supplemental item in ref. (Eguchi T et al., 2022, Cells 11).

A

```
Homo sapiens myeloid zinc finger 1 (MZF1), transcript variant 2, mRNA
NCBI Reference Sequence: NM_198055.2   2666 bp    mRNA

  841 atgcaggaat caccactggg cctgcaggtg aaagaggagt cagaggttac agaggactca
                             ---------F1---------->
  901 gatttcctgg agtctgggcc tctagctgcc acccaggagt ctgtacccac cctcctgcct
   Ex3||Ex4
  961 gaggaggccc agagatgtgg gaccgtgctg gaccagatct ttccccacag caagactggg
                Ex4||Ex5                      <---------R1----------
```

B

```
Homo sapiens SCAN domain containing 1 (SCAND1), transcript variant 3, mRNA
NCBI Reference Sequence: NM_001385710.1   1732 bp    mRNA

  841 cgccgacctg gacgcagaga agccagagac ttcgcttcc ggctgccgca ggcgcttcgc
                  --------F1----------->
  901 tggtgcaggt aagctccgca cactctcggc cggtcccgag tccgactccc tcaagggtga
  961 cgcgagctct gccctttaac cggaaacgtc tccctgctca ccccaccccc gcgcagacgc
 1021 agtgctgagc acacagctac cggacaaaga gtgacgcccg gagctggagt tatggcggct
                             <--------R1--------
```

C

```
Homo sapiens SCAND2 mRNA, complete cds   GenBank: AF229246.1

  421 gagtgcagtt gcaaagctct tctctgacct gaacaatggc tgtagctgtg gaccaacaaa
       Ex1                       5'-UTR||CDS
  481 tccagactcc ttcagtacaa gatctccaaa tagttaaact ggaagaagat tcccactggg
  541 agcaggaaat ttcccttcaa gggaattacc ctggaccaga gacatcctgc cagagctttt
                                        ----------F2--------->
  601 ggcatttccg ttaccaagaa gcatcacgac cccgagaggc cctcctccag ctccagaagc
                                                   ----------F1------->
  661 tctgttgtca gtggctaagg ccagagaagt gtacaaaaga gcagatcctg gagttgctgg
                                                <------R2------
  721 tcctagaaca gttccggact gtccttctcc aggagatcca gatctgggtc agacagcagc
      --R2--
  781 atccggagag tggagaggag gcagtggccc tggtggaaga cttgcagaaa gaacctggaa
      <----------R1--------
  841 gacagaggct ggagcctcgg gcgaggccgt ccggccgcac ccctcctgct cagctgcggt
   :          Ex1||Ex2
   :
 1261 gtccccgcct cgcgcgtccc cgggccggac aaaagccatg gctgcttgtt atccggtcaa
                                              ----------F3--------->
 1321 tggaacgggt tatgtacgtc atgttgataa tccaaatgga gacggaagac gtgtgaaatg
                                                             CDS||3'-UTR
 1381 tattacatta cgttaaagaa cgggatgcca aggtaagtgg aggtatactt cgaatttttc
 1441 tagaaggtaa agcctagttt gctgacattg aacccaaatt tgatagactg ctgtttttct
 1501 ggtctgacca tcgcaaccct catgaagtac aaccagcata tgctacaaag tacgcaataa
            <----------R3---------
```

D

```
Homo sapiens SCAN domain containing 2 pseudogene (SCAND2P), transcript variant 1, non-coding RNA
NCBI Reference Sequence: NR_004859.1   4509 bp    RNA

  541 ttcaagggaa ttaccctgga ccagagacat cctgccagag cttttggcat ttccgttacc
                             --------F2---------->
  601 aagaagcatc acgaccccga gaggccctcc tccagctcca gaagctctgt tgtcagtggc
                             --------F1---------->
  661 taaggccaga gaagtgtaca aaagagcaga tcctggagtt gctggtccta gaacagttcc
                                        <---------R2---------
  721 cgactgtcct tctccaggag atccagatct gggtcagaca gcagcatccg gagagtggag
                                                  <-------R1-----------
  781 aggaggcagt ggccctggtg gaagacttgc agaaagaacc tggaagacag aggctggagc
   :   -----                                                  Ex1||Ex2
   :
 1261 gtccccgggc cggacaaaag ccatggctgc ttgttatccg gtcaatggaa cgggttatgt
                          ----------F3-------->
 1321 acgtcatgtt gataatccaa atggagacgg aagacgtgtg aaatgtatta cattacgtta
```

**Figure S2.** Primer design for human MZF1(ZSCAN6), SCAND1, and SCAND2(SCAND2P) gene sequences. (A) MZF1 transcript variant 2 mRNA: NM_198055.2. (B) SCAND1 transcript variant 3 mRNA: NM_001385710.1. (C) SCAN domain containing 2 pseudogene (SCAND2P) transcript variant 1 non-coding RNA: NR_004859.1. (D) SCAND2 mRNA complete CDS: AF229246.1.

**Table S1.** Primer sequences for qRT-PCR.

| Primer name | Sequences (5′ to 3′) | Refseq ID | Location | Position of the 5' of the primer |
|---|---|---|---|---|
| MZF1 h F1 | TGCAGGTGAAAGAGGAGTCA | NM_198055.2 | Exon 3 | 863 |
| MZF1 h R1 | AGTCTTGCTGTGGGGAAAGA | NM_198055.2 | Exon 5 | 1017 |
| SCAND1 h F1 | CGCAGAGAAGCCAGAGACTT | NM_001385710.1 | Exon 3 | 853 |
| SCAND1 h R2 | CGG GCG TCA CTC TTT GTC | NM_001385710.1 | Exon 3 | 1060 |
| SCAND2 h F1 | CCTCCTCCAGCTCCAGAAG | NR_004859 | Exon 1 | 626 |
| SCAND2 h R1 | CTCCTCTCCACTCTCCGGAT | NR_004859 | Exon 1 | 785 |
| SCAND2 h F2 | CCAGAGACATCCTGCCAGAG | NR_004859 | Exon 1 | 561 |
| SCAND2 h R2 | CTAGGACCAGCAACTCCAGG | NR_004859 | Exon 1 | 711 |
| SCAND2 h F3 | CATGGCTGCTTGTTATCCGG | AF229246.1 | CDS* | 1297 |
| SCAND2 h R3 | CTTCATGAGGGTTGCGATGG | AF229246.1 | 3′-UTR** | 1527 |
| HSP90AA1 h F1 | GAGCAGTACGCTTGGGAGTC | NM_001017963.3 | Exon 4 | 1183 |
| HSP90AA1 h R1 | TCCACGACCCATAGGTTCAC | NM_001017963.3 | Exon 5 | 1260 |
| RNA18SN5 hmr F1245 | GACTCAACACGGGAAACCTC | NR_003286.4 | n.a.*** | 1245 |
| RNA18SN5 hmr R1364 | AGACAAATCGCTCCACCAAC | NR_003286.4 | n.a.*** | 1364 |

*Coding DNA sequence. **3′-untranslated region, *** not applicable.

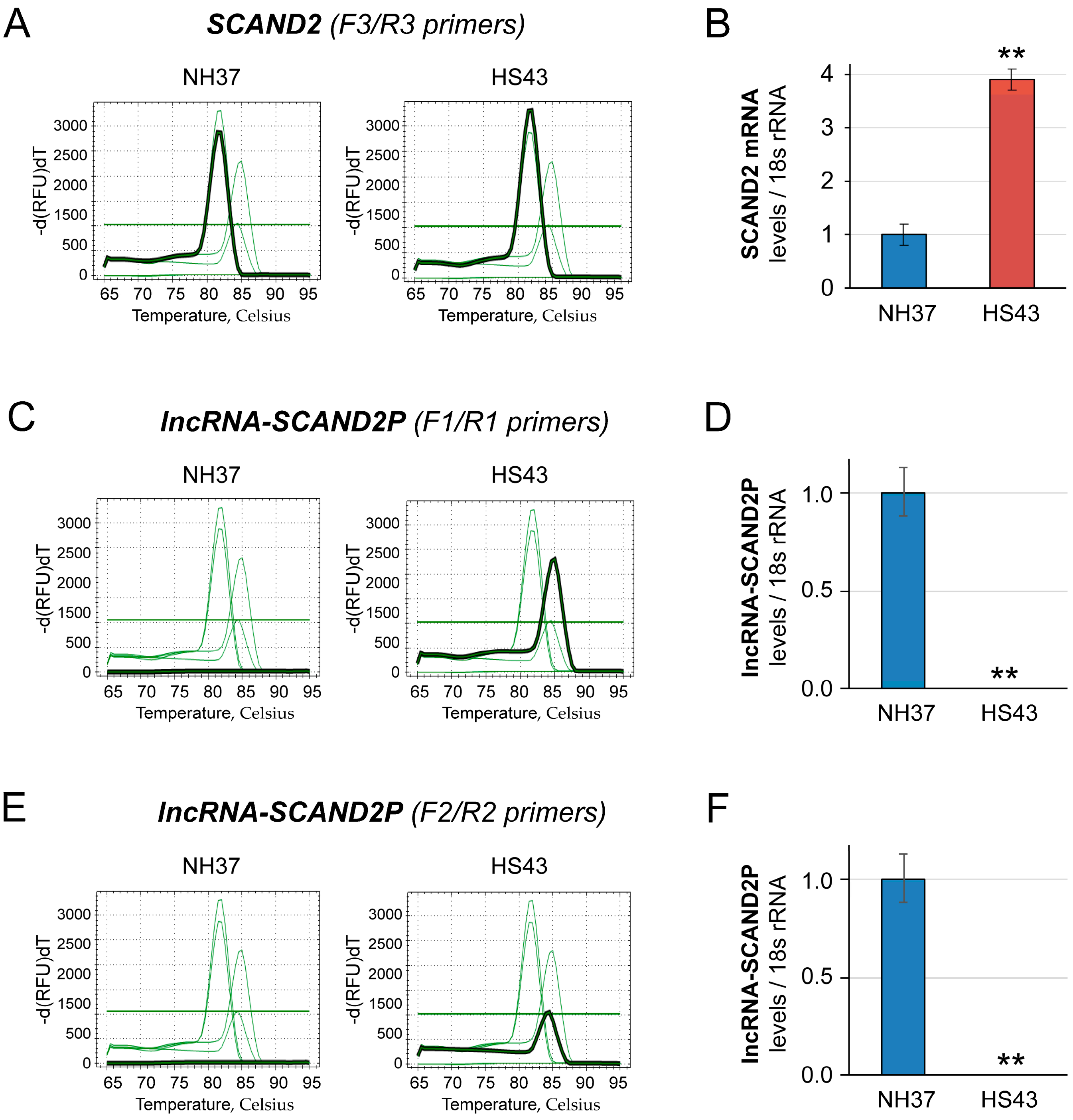


**Figure S3.** Melting curve analysis and qRT-PCR for SCAND2 mRNA and lncRNA-SCAND2P. We examined the correlation between heat shock stress (HSS) and the expression of SCAND2 mRNA (AF229246.1) (A, B) and its pseudogene variant lncRNA-SCAND2P (NR_004859.1) (C–F) in DU-145 prostate cancer cells. (A, C, E) Melting curves of PCR amplicons, indicating single amplicons in each PCR. (B, D, F) qRT-PCR analyses. (A, B) A region of the complete coding sequence of SCAND2 mRNA, using a primer pair (SCAND2 h F3 and R3), was upregulated with HSS. (C–F) Regions of the non-coding RNA transcript variant 1 of SCAND2P, using primer pairs (SCAND2 h F1/R1 (C, D) or SCAND2 h F2/R2 (E, F)) were detected under the non-heated condition but became under detection limit after HSS. **P < 0.01, n = 3.

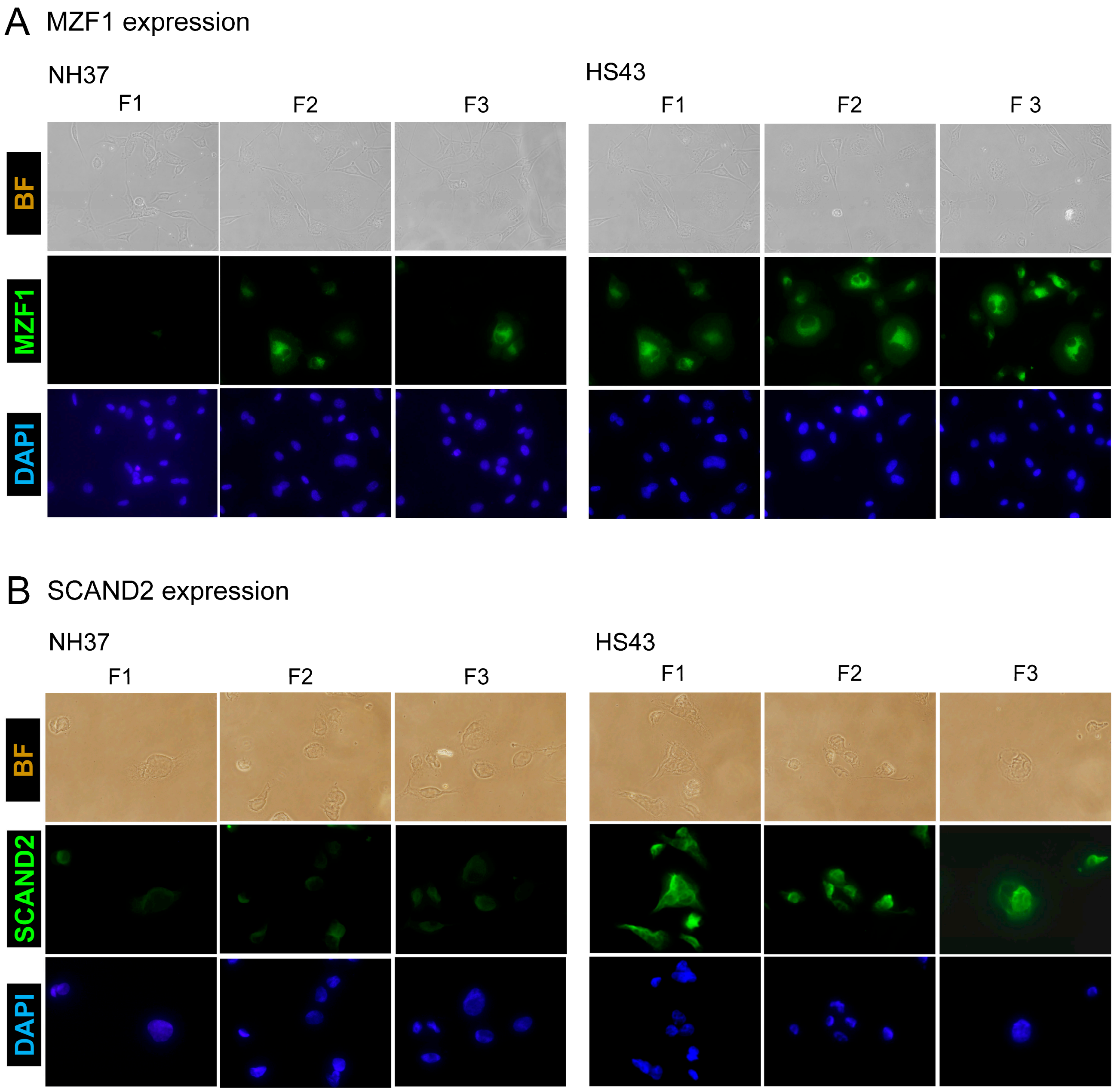


**Figure S4.** Immunocytochemistry for MZF1 (A) and SCAND2 (B) expression in DU-145 cells with or without heat shock. Cells were cultured under two conditions; non-heated at 37℃ (NH37) and heat-shocked at 43℃ (HS43) for 30 min. Three different fields (F1, F2, and F3) for each condition were shown. BF, bright field.

**Table S2.** Co-expression correlation of SCAN-TF genes vs. HSPs in prostate adenocarcinoma specimens.

| vs MZF1 | | | | vs SCAND1 | | | | vs SCAND2 | | | |
|---|---|---|---|---|---|---|---|---|---|---|---|
| Correlated Gene | Spearman's Correlation | p-Value | q-Value | Correlated Gene | Spearman's Correlation | p-Value | q-Value | Correlated Gene | Spearman's Correlation | p-Value | q-Value |
| HSPBP1 | 0.504 | 8.89E-33 | 2.82E-31 | HSPBP1 | 0.778 | 2.90E-100 | 1.81E-97 | HSPBP1 | 0.159 | 4.34E-04 | 1.54E-03 |
| ***HSPA13*** | -0.448 | 1.75E-25 | 3.29E-24 | ***HSPA13*** | -0.572 | 7.79E-44 | 1.05E-42 | ***HSPA13*** | -0.357 | 4.18E-16 | 9.13E-15 |
| ***HSPA4*** | -0.358 | 3.41E-16 | 2.70E-15 | ***HSPA4*** | -0.303 | 7.97E-12 | 2.47E-11 | ***HSPA4*** | -0.394 | 1.52E-19 | 4.80E-18 |
| HSPBAP1 | 0.354 | 6.99E-16 | 5.37E-15 | HSPBAP1 | 0.347 | 2.94E-15 | 1.09E-14 | HSPBAP1 | 0.281 | 2.62E-10 | 2.80E-09 |
| HSPB11 | 0.352 | 1.08E-15 | 8.16E-15 | HSPB11 | 0.516 | 1.36E-34 | 1.20E-33 | HSPB11 | 0.248 | 2.99E-08 | 2.35E-07 |
| ***HSPA4L*** | -0.326 | 1.58E-13 | 9.53E-13 | ***HSPA4L*** | -0.411 | 2.66E-21 | 1.32E-20 | HSPA4L | -0.0341 | 0.452 | 0.571 |
| ***HSP90AA1*** | -0.321 | 3.63E-13 | 2.11E-12 | HSP90AA1 | -0.188 | 2.86E-05 | 5.87E-05 | ***HSP90AA1*** | -0.32 | 4.34E-13 | 6.62E-12 |
| ***HSPH1*** | -0.3 | 1.42E-11 | 6.96E-11 | HSPH1 | -0.191 | 2.20E-05 | 4.55E-05 | ***HSPH1*** | -0.312 | 1.67E-12 | 2.37E-11 |
| HSPA7 | 0.298 | 1.69E-11 | 8.21E-11 | HSPA7 | 0.241 | 7.45E-08 | 1.84E-07 | HSPA7 | 0.277 | 4.54E-10 | 4.67E-09 |
| HSPB9 | 0.278 | 4.05E-10 | 1.70E-09 | HSPB9 | 0.342 | 7.59E-15 | 2.76E-14 | HSPB9 | 0.269 | 1.64E-09 | 1.56E-08 |
| ***HSP90B3P*** | -0.246 | 3.47E-08 | 1.20E-07 | HSP90B3P | -0.403 | 1.78E-20 | 8.51E-20 | HSP90B3P | -0.242 | 6.29E-08 | 4.72E-07 |
| HSPE1 | 0.216 | 1.38E-06 | 3.98E-06 | HSPE1 | 0.392 | 2.07E-19 | 9.38E-19 | HSPE1 | -0.124 | 6.20E-03 | 0.0161 |
| HSPB8 | -0.21 | 2.79E-06 | 7.77E-06 | HSPB8 | -0.0333 | 0.463 | 0.528 | HSPB8 | 0.0593 | 0.191 | 0.293 |
| HSPB7 | -0.203 | 6.01E-06 | 1.61E-05 | HSPB7 | -0.0237 | 0.602 | 0.66 | HSPB7 | 0.0263 | 0.563 | 0.672 |
| HSPA12A | -0.203 | 6.43E-06 | 1.72E-05 | HSPA12A | -0.149 | 9.59E-04 | 1.69E-03 | HSPA12A | 0.0673 | 0.138 | 0.225 |
| HSP90AB4P | 0.184 | 4.27E-05 | 1.03E-04 | HSP90AB4P | 0.189 | 2.55E-05 | 5.25E-05 | HSP90AB4P | 0.149 | 9.91E-04 | 3.21E-03 |
| HSPA1B | 0.176 | 9.23E-05 | 2.14E-04 | HSPA1B | 0.274 | 7.90E-10 | 2.19E-09 | HSPA1B | -0.0374 | 0.41 | 0.531 |
| ***HSPA5*** | -0.174 | 1.11E-04 | 2.54E-04 | HSPA5 | -0.212 | 2.41E-06 | 5.40E-06 | HSPA5 | -0.29 | 6.94E-11 | 8.09E-10 |
| ***HSPA8*** | -0.157 | 5.07E-04 | 1.06E-03 | HSPA8 | -0.352 | 1.07E-15 | 4.07E-15 | HSPA8 | -0.274 | 7.08E-10 | 7.10E-09 |
| HSPG2 | -0.151 | 7.93E-04 | 1.62E-03 | HSPG2 | -0.282 | 2.42E-10 | 6.88E-10 | HSPG2 | -0.0246 | 0.587 | 0.693 |
| ***HSP90AB2P*** | -0.15 | 8.69E-04 | 1.76E-03 | HSP90AB2P | -0.268 | 1.92E-09 | 5.20E-09 | HSP90AB2P | -0.202 | 6.80E-06 | 3.55E-05 |
| HSPA6 | 0.145 | 1.36E-03 | 2.69E-03 | HSPA6 | 0.163 | 2.94E-04 | 5.49E-04 | HSPA6 | 0.0642 | 0.157 | 0.25 |
| HSPA1A | 0.137 | 2.49E-03 | 4.72E-03 | HSPA1A | 0.279 | 3.51E-10 | 9.90E-10 | HSPA1A | -0.0836 | 0.0651 | 0.121 |
| HSPB6 | -0.134 | 2.95E-03 | 5.54E-03 | HSPB6 | 0.102 | 0.0244 | 0.0364 | HSPB6 | 0.0603 | 0.183 | 0.283 |
| HSPA14 | -0.117 | 9.41E-03 | 0.0163 | HSPA14 | -0.277 | 5.08E-10 | 1.42E-09 | HSPA14 | -0.0762 | 0.0928 | 0.163 |
| HSP90B1 | -0.112 | 0.0132 | 0.0223 | HSP90B1 | -0.167 | 2.04E-04 | 3.87E-04 | HSPB1 | 0.034 | 0.454 | 0.572 |
| HSPB3 | -0.112 | 0.0137 | 0.023 | HSPB3 | 0.0262 | 0.563 | 0.625 | HSPB3 | -0.0508 | 0.263 | 0.377 |
| HSPA1L | 0.0931 | 0.0397 | 0.0615 | HSPA1L | 0.0935 | 0.039 | 0.0564 | HSPA1L | 0.156 | 5.28E-04 | 1.84E-03 |
| ***HSP90AB1*** | -0.0746 | 0.0996 | 0.142 | HSP90AB1 | -0.11 | 0.0155 | 0.0238 | HSP90AB1 | -0.276 | 5.67E-10 | 5.76E-09 |
| HSPB2 | -0.0639 | 0.159 | 0.215 | HSPB2 | 0.177 | 8.44E-05 | 1.66E-04 | HSPB2 | 0.109 | 0.0159 | 0.0366 |
| HSPB1 | 0.0573 | 0.207 | 0.271 | HSPB1 | 0.45 | 9.36E-26 | 5.66E-25 | HSP90B1 | -0.244 | 4.93E-08 | 3.74E-07 |
| HSPA9 | -0.0491 | 0.279 | 0.351 | HSPA9 | -0.208 | 3.71E-06 | 8.17E-06 | HSPA9 | -0.246 | 3.54E-08 | 2.76E-07 |
| HSPA2 | -0.042 | 0.354 | 0.431 | HSPA2 | 0.204 | 5.40E-06 | 1.18E-05 | HSPA2 | -0.0254 | 0.576 | 0.684 |
| HSPD1 | -0.0358 | 0.43 | 0.508 | HSPD1 | -0.0162 | 0.721 | 0.767 | HSPD1 | -0.26 | 5.75E-09 | 5.05E-08 |
| HSPB1P1 | 0.0191 | 0.675 | 0.736 | HSPB1P1 | 0.406 | 9.63E-21 | 4.65E-20 | HSPB1P1 | 0.0196 | 0.665 | 0.757 |
| HSPA12B | 0.0161 | 0.723 | 0.778 | HSPA12B | 0.0806 | 0.0754 | 0.104 | HSPA12B | 0.135 | 2.77E-03 | 7.98E-03 |

Spearman's correlation < -0.25 were shown in red. p-Values < 1E-10 were shown in yellow. q-Values < 1E-10 were shown in green.

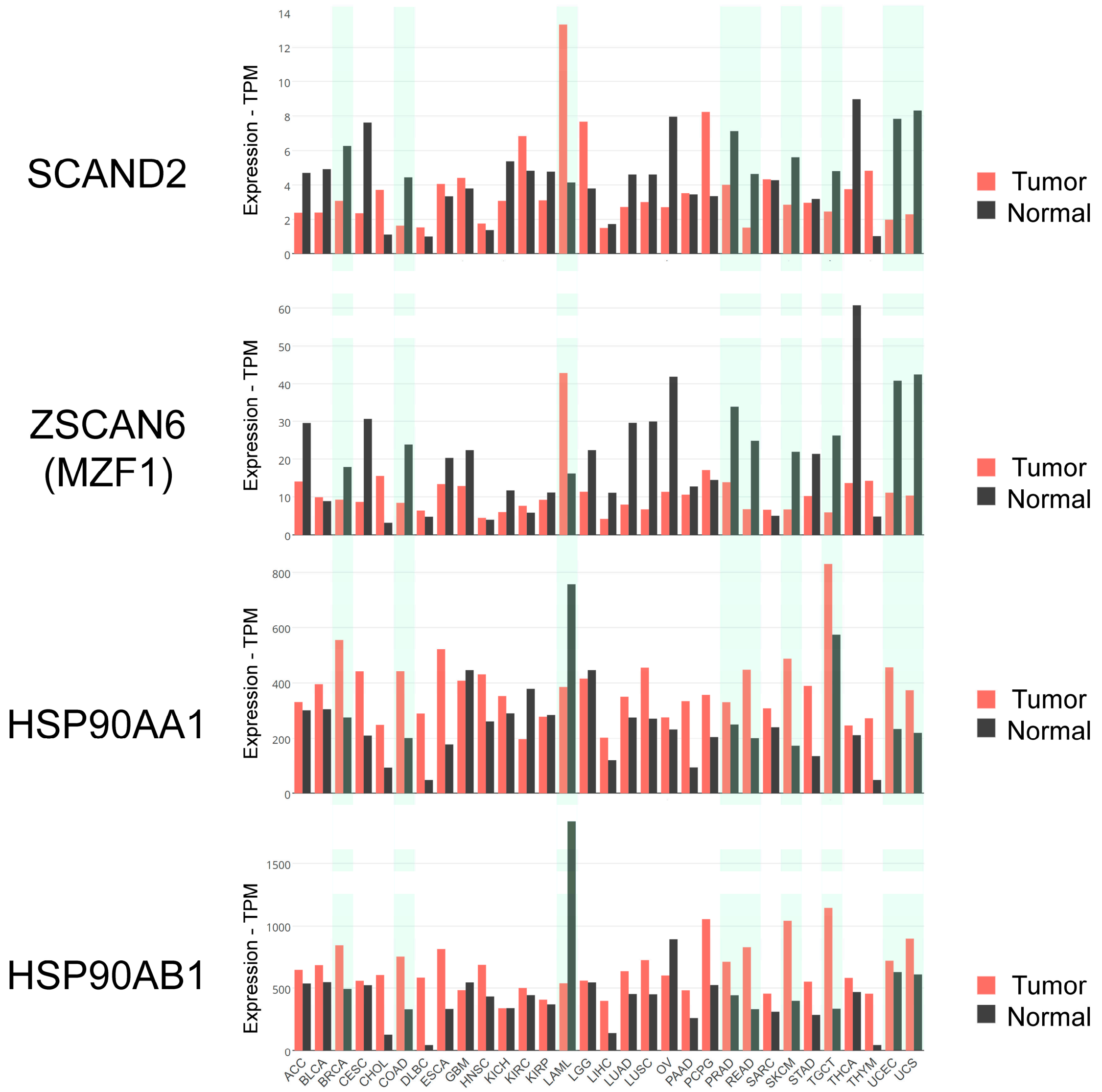


**Figure S5.** Gene expression profiling of SCAND2, ZSCAN6(MZF1), and HSP90 genes in various tumor types vs. paired normal tissues. Data were from TCGA PanCancer Atlas.

**Table S3.** Patients-derived tumor vs. normal tissue samples from TCGA PanCancer Atlas and GTEx data

| TCGA | Detail | Tumor | Normal | GTEx | Num |
|---|---|---|---|---|---|
| ACC | Adrenocortical carcinoma | 77 | - | Adrenal Gland | 128 |
| BLCA | Bladder Urothelial Carcinoma | 404 | 19 | Bladder | 9 |
| BRCA | Breast invasive carcinoma | 1085 | 112 | Breast | 179 |
| CESC | Cervical squamous cell carcinoma and endocervical adenocarcinoma | 306 | 3 | Cervix Uteri | 10 |
| CHOL | Cholangio carcinoma | 36 | 9 | - | - |
| COAD | Colon adenocarcinoma | 275 | 41 | Colon | 308 |
| DLBC | Lymphoid Neoplasm Diffuse Large B-cell Lymphoma | 47 | - | Blood | 337 |
| ESCA | Esophageal carcinoma | 182 | 13 | Esophagus | 273 |
| GBM | Glioblastoma multiforme | 163 | - | Brain | 207 |
| HNSC | Head and Neck squamous cell carcinoma | 519 | 44 | - | - |
| KICH | Kidney Chromophobe | 66 | 25 | Kidney | 28 |
| KIRC | Kidney renal clear cell carcinoma | 523 | 72 | Kidney | 28 |
| KIRP | Kidney renal papillary cell carcinoma | 286 | 32 | Kidney | 28 |
| LAML | Acute Myeloid Leukemia | 173 | - | Bone Marrow | 70 |
| LGG | Brain Lower Grade Glioma | 518 | - | Brain | 207 |
| LIHC | Liver hepatocellular carcinoma | 369 | 50 | Liver | 110 |
| LUAD | Lung adenocarcinoma | 483 | 59 | Lung | 288 |
| LUSC | Lung squamous cell carcinoma | 486 | 50 | Lung | 288 |
| MESO | Mesothelioma | 87 | - | - | - |
| OV | Ovarian serous cystadenocarcinoma | 426 | - | Ovary | 88 |
| PAAD | Pancreatic adenocarcinoma | 179 | 4 | Pancreas | 167 |
| PCPG | Pheochromocytoma and Paraganglioma | 182 | 3 | - | - |
| PRAD | Prostate adenocarcinoma | 492 | 52 | Prostate | 100 |
| READ | Rectum adenocarcinoma | 92 | 10 | Colon | 308 |
| SARC | Sarcoma | 262 | 2 | - | - |
| SKCM | Skin Cutaneous Melanoma | 461 | 1 | Skin | 557 |
| STAD | Stomach adenocarcinoma | 408 | 36 | Stomach | 175 |
| TGCT | Testicular Germ Cell Tumors | 137 | - | Testis | 165 |
| THCA | Thyroid carcinoma | 512 | 59 | Thyroid | 278 |
| THYM | Thymoma | 118 | 2 | Blood | 337 |
| UCEC | Uterine Corpus Endometrial Carcinoma | 174 | 13 | Uterus | 78 |
| UCS | Uterine Carcinosarcoma | 57 | - | Uterus | 78 |
| UVM | Uveal Melanoma | 79 | - | - | - |

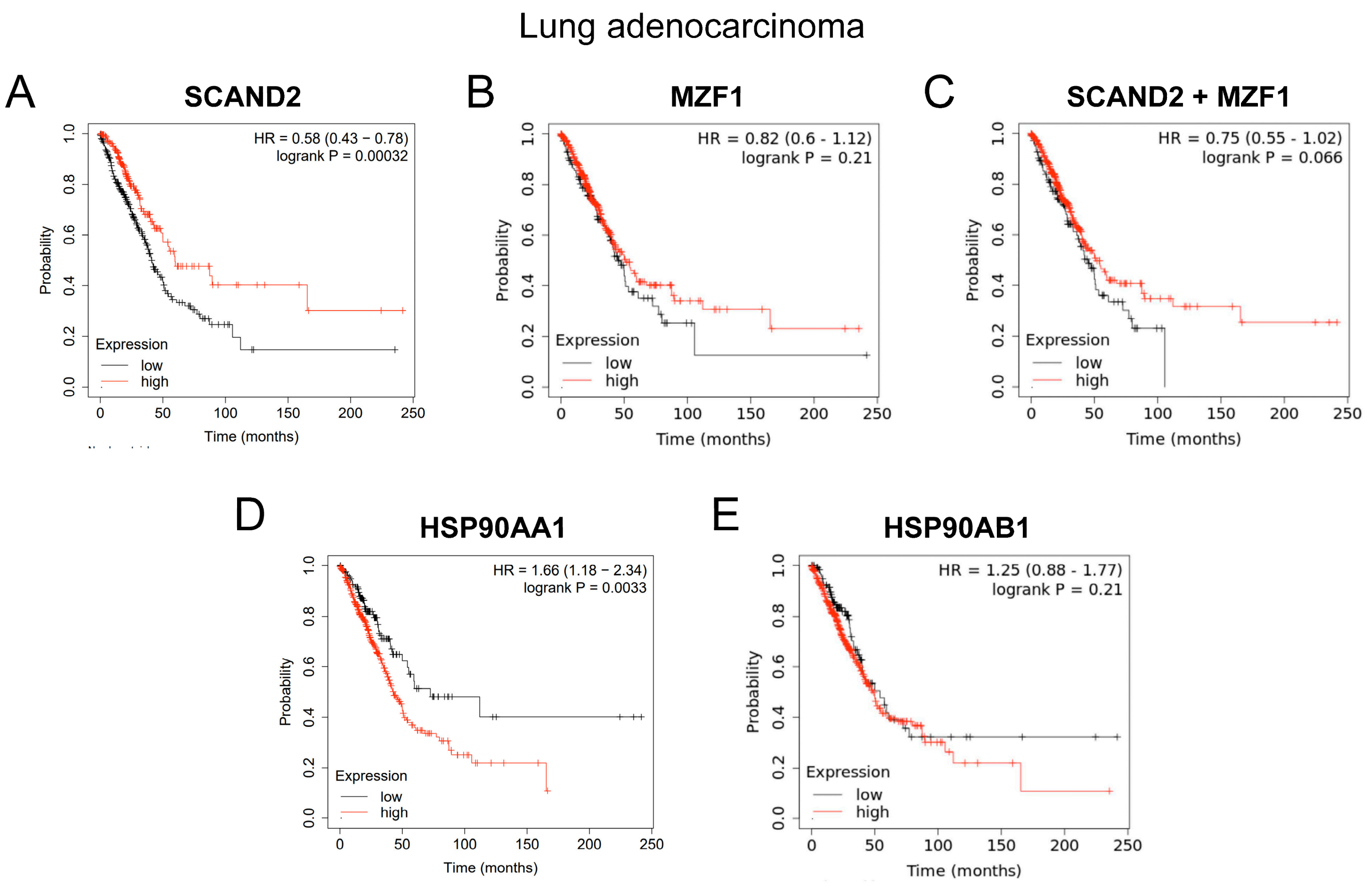


**Figure S6.** Kaplan-Meier analysis of SCAN-TFs and HSP90 expression in lung adenocarcinoma. Data were from TCGA PanCancer Atlas, lung adenocarcinoma (LUAD), n=504. SCAND2 and MZF1 expression are correlated with a better prognosis, whereas HSP90 expression is correlated with a poor prognosis of lung adenocarcinoma.
